# Multiple uses of dexmedetomidine in small animals: a mini review

**DOI:** 10.3389/fvets.2023.1135124

**Published:** 2023-06-05

**Authors:** Chiara Di Franco, Flavia Evangelista, Angela Briganti

**Affiliations:** ^1^Department of Veterinary Sciences, University of Pisa, Pisa, Italy; ^2^Vet Hospital H24, Firenze, Italy

**Keywords:** dexmedetomidine, veterinary, sedation, analgesia, hemodynamics

## Abstract

Dexmedetomidine is an alpha-2 adrenergic agonist, which use had an exponential increase in human and veterinary medicine in the last 10 years. The aim of this mini review is to summarize the various uses of dexmedetomidine underlining its new applications and capabilities in the small animals’ clinical activity. While this drug was born as sedative in veterinary medicine, some studies demonstrated to be effective as an analgesic both in single administration and in continuous infusion. Recent studies have also shown the role of dexmedetomidine as an adjuvant during locoregional anesthesia, increasing the duration of the sensitive block and consequently decreasing the demand for systemic analgesics. The various analgesic properties make dexmedetomidine an interesting drug for opioid-free analgesia. Some studies highlighted a potential neuroprotective, cardioprotective and vasculoprotective role of dexmedetomidine, thus conferring it a place in critical care medicine, such as trauma and septic patients. Dexmedetomidine has demonstrated to be a multitasking molecule and it is ready to face new challenges.

## Graphical Abstract

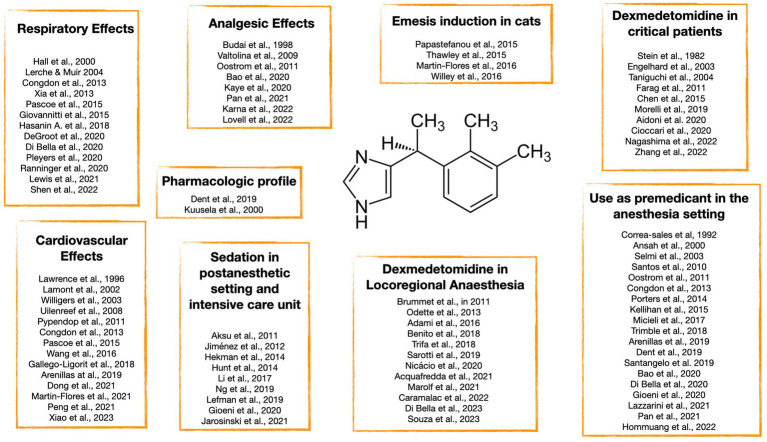

## Introduction

Dexmedetomidine, the dextrorotatory enantiomer of medetomidine, is an alpha-2 adrenergic agonist drug. The sedative properties, combined with analgesic effects and minimal influence on respiratory drive, and unique pharmacological profile, make dexmedetomidine suitable for use in many clinical scenarios (1–4). The distinctive features of dexmedetomidine allow wide perioperative applications of the drug. While traditionally considered solely as a sedative in veterinary medicine, evidence in the literature supports the use of dexmedetomidine as a pre-anesthetic agent (5, 6) showing a remarkable sparing effect on anesthetics, as perioperative analgesic (7, 8), and as adjuvant in locoregional anesthesia (9).

Current evidence in human medicine also suggests its potential benefit in special subsets of patients, thanks to hemodynamic, renal and neuronal protective effects (10, 11).

The aim of this paper is to present several facets of dexmedetomidine and to explore the current and new clinical applications in small animal veterinary medicine.

## Pharmacologic profile

Dexmedetomidine is a highly selective alpha-2 agonist with sedative and analgesic properties.

When administered intravenously, at the dose of 5 mcg/kg, the onset is about 1 min in dogs (12).

Maximal sedation is obtained between 10 and 20 min, when dexmedetomidine is administrated intravenously at 10–20 mcg/kg in dogs (13).

## Respiratory effects

An experimental study in dogs demonstrated that following dexmedetomidine administration the tidal volume (Vt), measured by electrical impedance tomography, may increase, while a decrease in the respiratory rate occurs (4). In the same study the authors evidenced a characteristic breathing pattern with multiple breaths followed by a period of apnea after dexmedetomidine administration. On the contrary, a previous study found a decrease in Vt, respiratory rate and minute ventilation after medetomidine injection in dogs (14). The discrepancy, however, could be due to the different methods of measuring Vt: in fact, the use of a pneumotachograph connected to a tight face mask could have increased the resistance to gas flow, thus underestimating Vt.

Another study confirmed the absence of respiratory changes in dogs premedicated intramuscularly with 10 mcg/kg of dexmedetomidine and maintained with a continuous infusion at 0.5 mcg/kg/h (15). Low doses of dexmedetomidine (1 mcg/kg/h) CRI have been shown to improve oxygenation and respiratory system compliance, reducing intrapulmonary shunt fraction and airway resistances (3). The results of this study agree with similar studies conducted in human medicine (16, 17) in which dexmedetomidine improved oxygenation, decreased intrapulmonary shunts and ameliorated respiratory mechanics.

A recent review of the literature (18) emphasizes the use of a sedative, indicating dexmedetomidine as an option, in laryngeal function evaluation protocols in dogs. According to the authors, administration of dexmedetomidine, prior to an induction agent, provides good laryngeal examination conditions and it is one of the best options to preserve laryngeal motility. In contrast, induction without prior sedation, may suppress laryngeal movements impairing laryngeal evaluation. Another study shows that two dogs out of eight had a false positive result for laryngeal dysfunction with propofol; the same dogs, sedated with dexmedetomidine had a normal laryngeal function evaluation (2).

At current times, numerous studies in human and veterinary medicine agree that the use of dexmedetomidine does not lead to significant alterations on the respiratory function (2–4, 19–22).

## Use as premedicant in the anesthesia setting

Dexmedetomidine exerts its sedative effects by acting on the locus coeruleus (LC) of the midbrain where it inhibits the release of neurotransmitters from the synaptic terminals, preventing neuronal signaling and reducing the state of consciousness (23). LC has been linked to a variety of physiological regulatory processes, including sleep and wake regulation, nociception, and orientation (24). Since its discovery, this molecule has been widely used in clinical practice as a sedative drug alone or in combination with opioids (5, 6, 25). The doses used in cats and dogs are highly variable depending on the route of administration. Based on data collected from a systematic review conducted in 2021, doses ranged from 1 to 10 mcg/kg IV or IM (26). Combining dexmedetomidine with other drugs has reported to produce greater sedation than the single administration (27, 28).

Dexmedetomidine has also been used in continuous rate infusion (CRI), both for its analgesic and sedative properties; the most frequently reported dosage is from 0.5 to 1 mcg/kg/h (3, 15, 29, 30).

The intranasal route for dexmedetomidine administration as an alternative to its classic intramuscular or intravenous administration has been investigated in dogs and cats (31–33). Results in dogs showed higher sedation scores with intranasal administration in comparison to intramuscular; central absorption of the drug through the turbinates, could be an explanation for this result (31). On the contrary, in cats, intranasal administration of dexmedetomidine at 20 mcg/kg produced moderate sedation accompanied to vomiting and hyperthermia (33).

Oral transmucosal route has been evaluated both in dogs and cats, with positive results; in dogs an effective sedation was obtained with 20 mcg/mg of dexmedetomidine alone (12), or with 10 mcg/kg of dexmedetomidine combined with 0.4 mg/kg of methadone (34). In cats, doses of 20 and 40 mcg/kg were used in combination with 20 mcg/kg of buprenorphine (35, 36) and the higher dose produced a similar level of sedation and analgesia compared to intramuscular administration (36).

The use of dexmedetomidine as sedative is a bedrock in the clinical setting, but the dosages are slightly changed in the last years, and overall, the continuous infusion opened a new scenario: the possibility to maintain sedation with limited cardiovascular effects. New routes of administration have been studied which can be an interesting alternative to the conventional methods used in the routine practice.

## Sedation in postanaesthetic setting and intensive care unit

Dexmedetomidine has taken more and more ground in regard to its use in human intensive care; its inhibitory activity at the level of the locus coeruleus determines a depression of the CNS much more similar to the physiological sleep–wake cycle compared to other molecules (i.e., propofol, benzodiazepines, trazodone) reducing the incidence of delirium and the length of hospitalization as well as the duration of mechanical ventilation (37).

Delirium has not been defined yet in veterinary medicine and it is preferrable to talk about “psychogenic stress” during hospitalization in intensive care unit (38, 39).

Post anesthesia dysphoria, which could be the same of emergence delirium, is quite common in veterinary medicine; the mechanism by which this occurs is not fully clarified and the following can be listed as possible causes: use of inhaled anesthetics, opioids and some injectable anesthetics.

The small animal literature reports few studies conducted on the use of alpha-2 agonist during recovery. A bolus of 5 mcg/kg of medetomidine IV resulted efficacious as rescue sedation during recovery from general anesthesia in dogs (40), and administration of a bolus of 2 mcg/kg of dexmedetomidine in dogs prior to recovery from prolonged anesthesia, compared to no administration, results in a smoother recovery and better quality of recovery (41).

In a recent study dexmedetomidine 0.5 mcg/kg administered IV slowly before the end of inhalation anesthesia showed better recovery albeit significantly longer (42).

Dexmedetomidine has also been used as part of a multimodal approach to refractory status epilepticus in dogs (43), nevertheless it is important to consider that its role could be important for the muscular relaxation and not for the control of the seizure. In fact, in human medicine dexmedetomidine has been shown to be efficacious in association of general anesthetics for electroconvulsive therapy (44) and it is recommended for sedation in children for electroencephalography (EEG) because it has a minimum impact on EEG (45).

## Analgesic effects

Analgesic effects of dexmedetomidine are mediated in the dorsal horns of the spinal cord where it is thought to reduce the activity of ascending nociceptive neurons (23).

It is also reported that supraspinal structures as LC and periaqueductal gray (PAG) may be involved in dexmedetomidine mediated analgesia (46).

The analgesic effects of dexmedetomidine are well known and in human medicine researchers are evaluating the inclusion of dexmedetomidine in the enhanced recovery after surgery (ERAS) protocol for the perioperative pain management (47). In veterinary medicine a recent systematic review confirmed the efficacy and the safety when used in bolus in a balance anesthetic protocol (26). In dogs undergoing ovariohysterectomy dexmedetomidine (7 or 10 mcg/kg) and maropitant combined with a low dose of morphine (0.2 mg/kg) resulted in equivalent or better analgesia than morphine alone at a higher dose (0.6 mg/kg) (48).

The continuous infusion has been demonstrated to be effective more than 10 years ago with a clinical study in critically ill dogs; dogs that received dexmedetomidine infusion (25 mcg/m^2^/h) required less analgesic rescue compared to the control group (treated with morphine) and were calmer and more relaxed (7). A more recent clinical study compared the analgesic efficacy of a multimodal protocol based on infusion of ketamine plus dexmedetomidine to fentanyl infusion and the results showed a similar analgesic efficacy of the two protocols (49).

An experimental study in which auditory and somatosensory evoked potentials have been evaluated demonstrated that sedation appears at dosage of 1 mcg/kg/h while analgesia requires dosage of 3 mcg/kg/h (30). This means that clinical signs of sedation are not always associated with analgesia, and even though in the clinical setting it is rare that dexmedetomidine is the only analgesic used, this information is important for a correct use.

## Cardiovascular effects

Dexmedetomidine, as all the alpha-2 agonist, determines a peripheral vasoconstriction which consequently leads to a reflex bradycardia. Cardiovascular effects at doses commonly used in veterinary practice include a decrease in cardiac output, heart rate, sympathetic tone, and an increase in afterload due to increases in systemic vascular resistance, which may result in an increase in systemic and occasionally pulmonary pressures (21, 50).

In human patients it reduces opioids demand and hemodynamic variations (51). Furthermore, after an initial vasoconstriction the effect of dexmedetomidine changes completely causing a vasodilation (probably activating the endothelial muscle cells) which makes subsequent hypotension possible (51). On the contrary, vasodilation following the administration of dexmedetomidine, has not been reported in veterinary medicine.

The hemodynamic effects of dexmedetomidine in animals such as bradycardia and vasoconstriction with consequent hypertension have nowadays been acknowledged (25, 50). Moreover, when administered in continuous infusion at low doses (0.5–1 mcg/kg/h) the hemodynamic effects are much less relevant (15). Side effects such as 2nd degree AV block type II have been reported with dexmedetomidine infusion at 3 mcg/kg/h (52) due to reduction of the sympathetic tone. The reduction of the sympathetic tone produces the decrease of cardiac oxygen requirement suggesting a cardioprotective role of dexmedetomidine infusion (53). An experimental study in dogs demonstrated that myocardial energy requirement decreases with 1 mcg/kg of dexmedetomidine, whereas moderate coronary vasoconstriction occurs after 10 mcg/kg (54).

While for years dexmedetomidine has been contraindicated in veterinary patients with cardiac problems, nowadays in human medicine it is widely used for perioperative sedation in patients undergoing cardiac surgery (55–57). In dogs dexmedetomidine can increase valvular regurgitation and for this reason it has been indicated to be used with caution in patients with valvular regurgitation (58). On the other hand, possible beneficial effects have been reported in cats with concentric hypertrophic forms with dynamic left ventricular outflow tract obstruction (59).

In veterinary medicine the use of dexmedetomidine in these patients is slowly changing and a retrospective study demonstrated that dexmedetomidine was efficacious in reducing the use of vasopressors and antimuscarinics during pulmonic balloon valvuloplasty in dogs (60).

The cardiovascular effects of dexmedetomidine produce a blunt of the sympathetic response resulting in a stabilization of the hemodynamics in the perioperative period thus leading to a reduced stress for the cardiovascular system, and cardiopathic patients may benefit more than healthy patients.

## Dexmedetomidine in critical patients

Dexmedetomidine seems to play a protective role against injury in various organs. A study demonstrated the protective effect of dexmedetomidine in rats in which cerebral ischemia was induced. The authors supposed that dexmedetomidine may increase the concentration of anti-apoptotic proteins Bcl-2 (61). It is also known that catecholamines can have a direct toxic effect on neuronal tissue, therefore, dexmedetomidine acting as heteroreceptor causes a decrease in the release of epinephrine and norepinephrine thus decreasing the toxic effects (62, 63).

In a study in which endotoxemia was induced in rats following injection with *Escherichia coli* dexmedetomidine inhibited the inflammatory response and reduced the mortality rate (64).

An experimental study showed how the administration of dexmedetomidine reduced the production of cytokines and the mortality in rats subjected to cecum ligation and puncture (65).

In an experimental study conducted on swine, dexmedetomidine given at early stages of sepsis, exerted a beneficial effect on cardiac control and optimization of pulmonary artery pressure, while the deterioration of systemic hemodynamics was transient and reversible (66).

Dexmedetomidine has been used in septic human patients in the intensive care unit resulting in a decrease in the demand for vasoactive drugs such as norepinephrine (67).

Morelli et colleagues demonstrated that using dexmedetomidine for the sedation of septic patients guarantees a comparable level of sedation and reduces the dosage of norepinephrine required compared to propofol (68), and from a recent meta-analysis resulted that dexmedetomidine, in human patients with sepsis, could significantly reduce mortality compared with benzodiazepines but not with propofol (69).

It could be interesting to evaluate this aspect in veterinary medicine since currently, according to the authors’ knowledge, there is only one clinical study conducted on canine septic patients. In this study dexmedetomidine in CRI at a dosage of 3 mcg/kg/h was compared to fentanyl in dogs with pyometra, the authors concluded that dexmedetomidine could be used during general anesthesia of septic dogs without causing microcirculatory and hemodynamic impairment (70).

The low impact on the respiratory system, the positive effects on the cardiovascular system and the potential benefits on toxics effects associated to the well-known sedative and analgesic effects promote dexmedetomidine as unique and incomparable and may play a pivotal role in critical and septic patients.

## Dexmedetomidine in locoregional anesthesia

Locoregional anesthesia techniques are increasingly widespread and used for the treatment of intra and post-operative pain in veterinary medicine. The duration of the nerve block depends on the drug and the concentration chosen. The prolongation of the life of peripheral blocks over the years has been made possible using different molecules such as opioids, alpha-2 agonists, epinephrine, phenylephrine, ketamine and magnesium sulfate (9, 71). The ideal adjuvant should increase the sensory block and limiting the increase in motor block as much as possible to decrease the pain of the animal without affecting its ability to move. Sarotti et al. showed how a CRI of dexmedetomidine in dogs anaesthetized with isoflurane supplies a longer period without rescue analgesia without prolonging the duration of the motor block. The mechanism by which dexmedetomidine could increase the duration of the block is not entirely clear. Brummett et al. suggested that the addition of dexmedetomidine to a local anesthetic could cause the blockade of the hyperpolarization-activated cation (Ih) current (72). A human medicine review underlines how the direct effect on alpha-2 adrenergic receptors can cause vasoconstriction therefore the absorption of AL with prolongation of clinical effects (73); in veterinary medicine it is reported that vasoconstriction induced by dexmedetomidine (1 mcg/kg) may delay the systemic absorption of the local anesthetic (74) in the same way of epinephrine at 2 mcg/kg. Acquafredda et al., evaluated the effects of dexmedetomidine administered perineurally (0.15 mcg/kg) or intramuscularly (0.3 mcg/kg) on sensory and motor function and postoperative analgesia produced by lidocaine for sciatic and femoral nerve blocks in dogs. They showed that dexmedetomidine administered in combination with lidocaine resulted in an increase in the duration of sensory block and reduced the request for analgesia rescue in the immediate postoperative period for both perineural and systemic administrations (75). The addition of dexmedetomidine (1 mcg/kg) to ropivacaine 0.5% for sciatic and saphenous nerve blocks in dogs causes an increase in sciatic, fibular, tibial, and saphenous nerve sensory blockade duration without increasing the duration of motor blockade or proprioception deficits (76). Another study showed that the adjunct of 0.5 mcg/kg of dexmedetomidine to bupivacaine 0.5% for the block of sciatic and femoral nerves for knee surgery in dogs produced analgesia up to 24 h (77). The use of intraperitoneal dexmedetomidine (4 mcg/kg) in addition to ropivacaine for postoperative analgesia in cats undergoing ovariectomy has given unsatisfactory results: even though cats of dexmedetomidine plus ropivacaine group required rescue analgesia 4 h later compared to the ropivacaine group, no statistically significant differences were found, while a significant decrease in heart rate was revealed in the group with dexmedetomidine (78). Therefore, as stated by the authors, a significant difference was not highlighted due to the small number of samples.

Epidural use of dexmedetomidine has been evaluated in clinical studies in dogs (79–81) and all demonstrated its efficacy, the main problem is the eventual neurotoxic effect that has not been assessed (79).

The works regarding the use of dexmedetomidine during loco-regional anesthesia are limited both in human and veterinary medicine, therefore the extension of the studies on dexmedetomidine in this area would be desirable.

## Emesis induction in cats

One of the side effects of dexmedetomidine is vomit; this side effect is more pronounced in cats and the association with butorphanol and maropitant have been studied to reduce it (82, 83). Anyway, this effect can be favorable when there is the necessity to make cats vomit as apomorphine does not work in cats. In different studies dexmedetomidine has been proved to be effective in producing vomit in cats at a dosage between 6 and 18 mcg/kg IM (84, 85).

## Conclusion

Dexmedetomidine has been demonstrated to be a versatile molecule with several clinical applications other than the simple sedation and premedication. Its use is spreading from anesthesia to intensive care unit, because of numerous clinical uses. The sedative effect can be used to reduce postanesthetic delirium, and to manage hospitalization stress. Different route of administrations, such as intranasal and transmucosal, can facilitate the use in particular patients. The infusion can allow the maintenance of sedation, analgesia and muscular relaxation as long as needed with minimal cardiovascular impact. The analgesic properties confer to dexmedetomidine a pivotal role in multimodal analgesia, and it can play a role in locoregional anesthesia and in the opioid free protocols. The lack of negative respiratory impact, the stabilization of hemodynamics and the vasculoprotective effects can play a substantial role in the management of critical patients.

Right now, the use of this drug is still relegated to young and healthy patients, but it is time to move forward. There is sufficient evidence to say that the choice of dose to achieve the possible desired effects without the appearance of unintended effects is still unclear in the veterinary literature. The real challenge will be to promote studies in dogs and cats to investigate and identify the doses related to a positive or negative impact on outcomes in our critical patients, therefore further studies are desirable in order to get the most out of the use of this drug.

## Author contributions

CDF and AB contributed to conception and design of the study. CDF and FE wrote the first draft of the manuscript. All authors contributed to manuscript revision, read, and approved the submitted version.

## Conflict of interest

The authors declare that the research was conducted in the absence of any commercial or financial relationships that could be construed as a potential conflict of interest.

## Publisher’s note

All claims expressed in this article are solely those of the authors and do not necessarily represent those of their affiliated organizations, or those of the publisher, the editors and the reviewers. Any product that may be evaluated in this article, or claim that may be made by its manufacturer, is not guaranteed or endorsed by the publisher.
